# Preparation and Adsorption Performance Study of Graphene Quantum Dots@ZIF-8 Composites for Highly Efficient Removal of Volatile Organic Compounds

**DOI:** 10.3390/nano12224008

**Published:** 2022-11-14

**Authors:** Hao Li, Youliang Cheng, Jiaxian Li, Tiehu Li, Jia Zhu, Weibin Deng, Jiajia Zhu, Delong He

**Affiliations:** 1 School of Materials Science and Engineering, Northwestern Polytechnical University, Xi’an 710072, China; 2 Faculty of Printing, Packaging Engineering and Digital Media Technology, Xi’an University of Technology, Xi’an 710048, China; 3 Laboratoire de Mécanique Paris-Saclay, Université Paris-Saclay, CentraleSupélec, ENS Paris-Saclay, CNRS, 91190 Gif-sur-Yvette, France

**Keywords:** graphene quantum dot, ZIF-8, composite, volatile organic compounds, adsorption, toluene, ethyl acetate

## Abstract

Based on the large specific surface area and excellent adsorption potential of graphene quantum dots (GQDs) and zeolitic imidazolate framework-8 (ZIF-8) materials, a GQDs@ZIF-8 composite was constructed to achieve optimal matching of the microstructure and to acquire efficient adsorption of volatile organic compounds (VOCs). GQDs and ZIF-8 were synthesized and then compounded by the solution co-deposition method to obtain GQDs@ZIF-8 composites. GQDs were uniformly decorated on the surface of the ZIF-8 metal-organic framework (MOF), effectively restraining the agglomeration, improving the thermal stability of ZIF-8 and forming abundant active sites. Thus, the VOC removal percentage and adsorption capacity of the GQDs@ZIF-8 composites were significantly improved. Toluene and ethyl acetate were chosen as simulated VOC pollutants to test the adsorption performance of the composites. The results showed that, after the addition of GQDs, the adsorption property of GQDs@ZIF-8 composites for toluene and ethyl acetate was obviously improved, with maximum adsorption capacities of 552.31 mg/g and 1408.59 mg/g, respectively, and maximum removal percentages of 80.25% and 93.78%, respectively, revealing extremely high adsorption performance. Compared with raw ZIF-8, the maximum adsorption capacities of the composites for toluene and ethyl acetate were increased by 53.82 mg/g and 104.56 mg/g, respectively. The kinetics and isotherm study revealed that the adsorption processes were in accordance with the pseudo-first-order kinetic model and the Freundlich isotherm model. The thermodynamic results indicated that the adsorption process of the GQDs@ZIF-8 composites was a spontaneous, endothermic and entropy increase process. This study provides a new way to explore MOF-based adsorption materials with high adsorption capacity which have broad application prospects in VOC removal fields.

## 1. Introduction

At present, volatile organic compounds (VOCs) are increasingly generated in the field of industrial production, home decoration, coatings, etc., causing great harm to the environment and human health [1,2]. The removal and degradation of VOCs has become a key problem to be solved. The terminal treatment of VOCs mainly includes adsorption, combustion and other chemical methods. Among them, adsorption methods using activated carbon, molecular sieves and a variety of nanoporous materials are widely adopted due to their simplicity and convenient features [3,4,5]. However, these methods still have problems such as low adsorption capacity, ease of blockage and low thermal stability [6,7]. Therefore, ascertaining how to obtain new adsorption materials with high VOC adsorption capacity, high thermal stability and good regeneration performance is the focus of current research.

Metal-organic frameworks (MOFs) with high specific surface area and developed porosity are widely used in gas separation [8], water purification [9], catalyzation [10] and other fields. As a major MOF subfamily, zeolite imidazole framework-8 (ZIF-8) has attracted extensive interest from researchers due to its simple synthesis and high chemical and thermal stability [11,12,13,14]. Zhou et al. [15] prepared hybrid composites of ZIF-8 and graphene oxide (GO) in methanol at room temperature, showing their adjustable nanoscale morphology and porosity. Due to the synergistic effect between ZIF-8 and GO, the composite had a higher absorption capacity of VOCs than ordinary ZIF-8 crystals. When the GO content was 15 wt%, the maximum adsorption capacity of ZIF-8/GO for methylene chloride reached 240 mg/g. Qiu et al. [16] prepared ZIF-8 and its derived nitrogen-rich, porous carbon with different average sizes and proved that their specific surface area and pore volume decreased with increasing particle size. For porous carbon derived from ZIF-8, nitrogen-containing functional groups greatly improved the affinity for CO_2_ and VOCs. Meng et al. [17] prepared N-functionalized porous carbon (NFPC) with a high specific surface area from ZIF-8 and applied it to the adsorption and removal of gaseous acetone and toluene. NFPC-1000 had the best adsorption performance for acetone and toluene, and the equilibrium adsorption capacities were 203.4 mg/g and 297.1 mg/g, respectively. Song et al. [18] prepared ZIF-8 with methanol, deionized water and triethylamine (Et3N) mixed solution as the medium and modified ZIF-8 with sodium alginate (SA) and CaCl_2_ ZIF-8@CA, which greatly improved the adsorption property of Pb^2+^, and the maximum adsorption capacity was 1321.21 mg/g. Wang et al. [19] used ZIF-8 and fly ash (FA) to prepare a low-cost, small-volume and high-stability nanocomposite ZIF-8/FA, which was used to remove heavy metal ions in water, effectively solving the problems of low FA adsorption performance and difficult recovery of ZIF-8. In the field of aqueous solution treatment, current research is mainly focused on the combination of ZIF-8 with organic carriers and metal ions, while research on VOC adsorption still needs more attention to continuously improve VOC adsorption capacity. Thus, the adsorption of ZIF-8 material on various VOC pollutants needs to be deeply investigated to make full use of its excellent performance.

As a zero-dimensional carbon nanomaterial, graphene quantum dots (GQDs) have special luminous properties [20], low toxicity [21] and good biocompatibility [22,23]. They have good application value in photovoltaic cells, biological imaging and medical fields [24]. Meanwhile, their large specific surface area, good dispersion, rich active centers (edges, functional groups, dopants, etc.) and good adjustability of chemical and physical properties [25] also lay the foundation for their application in the adsorption field. GQDs also have the advantages of rich surface functional groups and many active sites [26]. They can improve the adsorption capacity for specific substances by functionalizing other specific functional groups. Mahmoud et al. [27] further modified rice-hull-based GQDs with barium hydroxide to prepare a GQDOs-Ba nanobiosorbent for the microwave-enhanced removal of Pb^2+^ and La^3+^ from water. The increase in GQDOs-Ba dosage improved the removal efficiency of Pb^2+^ and La^3+^ ions. Manna et al. [28] prepared a GQD-coated biological matrix for removing azo dyes, methylene blue, etc. Compared with GQDs, the removal capacity of the GQD-coated biological matrix was increased by 2–3 times. Nagaraj et al. [29] covalently linked GQDs to functional ionic liquids (IL) to prepare a highly stable IL-GQDs adsorbent for removing Cr^6+^ from contaminated water. An alkaline medium was used as the eluent for the regeneration study, and the removal efficiency of Cr6^+^ by IL-GQDs was still more than 80% after five cycles. Shao et al. [30] synthesized GQDs-TiO_2_ nanofilms by a hydrothermal method. The highest sensitivity was 13.8, and the response time was 18 s when detecting 50 ppm isopropanol gas at room temperature. Alivand et al. [31] prepared GQDs by embedding them in the self-assembly process of material of institute Lavoisier-101 (MIL-101) and obtained MIL-101@GQD composites. Compared with conventional MIL-101, MIL-101@GQD-3 showed 300.0% and 53.3% greater mesopore and total pore volumes, respectively, resulting in 1.7 and 2.8 times more benzene and toluene loading, respectively. At present, GQDs still have some shortcomings such as low stability and easy agglomeration. In addition, low-cost production and purification still have not been realized, which restricts the large-scale application of GQDs.

ZIF-8 has the advantages of structural adjustability and ultrahigh specific surface area. Both acidic and alkaline functional groups exist in its structures, which can adsorb pollutants with different properties. GQDs also have rich surface functional groups and abundant active sites, which can be grafted with other specific functional groups to improve the adsorption capacity for specific substances. However, GQDs easily agglomerate during the service process, which leads to the failure of the adsorbent. In the face of this shortage, new types of GQDs@ZIF-8 complex structures are expected to be designed. For this novel composite, GQDs were uniformly dispersed on the surface of ZIF-8 to achieve optimal matching of the microstructure, which takes synergetic advantage of GQDs and ZIF-8, improving the adsorption performance of the composite and further realizing the pH-value-regulated adsorption efficiency through the adjustment of surface functional groups. Based on this new concept, GQDs and ZIF-8 were prepared by the direct pyrolysis method and liquid-phase synthesis method, respectively, and then GQDs@ZIF-8 composites were synthesized by further combination of GQDs and ZIF-8 via the solution co-deposition method. Characterizations, including those by transmission electron microscope (TEM), X-ray diffractometer (XRD), X-ray photoelectron spectrometer (XPS), Fourier-transform infrared spectrometer (FT-IR), thermal gravimetric analyzer (TGA) and N_2_ adsorption-desorption, were used to investigate the morphology, crystal structure, components and pore structures of the composites. Toluene and ethyl acetate were chosen as simulated VOC pollutants to test the adsorption performance of the composites, revealing maximum adsorption capacities of 552.31 mg/g and 1408.59 mg/g, respectively. GQDs@ZIF-8 composites with high adsorption capacity were obtained in this work, which can be used for the highly efficient removal of VOCs.

## 2. Materials and Methods

### 2.1. Materials and Reagents

Citric acid (C_6_H_8_O_7_, purity ≥ 99.8%), used as the precursor material for preparing GQDs, was purchased from Tianjin Jiayu Fine Chemical Co., Ltd. (Tianjin, China). 2-Methylimidazole (C_4_H_6_N_2_, purity ≥ 98%) and zinc nitrate hexahydrate (Zn(NO_3_)_2_·6H_2_O, purity ≥ 99%), used as the precursor materials for preparing ZIF-8, were purchased from Beijing Boyu Gaoxin New Materials Co., Ltd. (Beijing, China) and Luoyang Haohua Chemical Reagent Co., Ltd. (Luoyang, China), respectively. Toluene (C_7_H_8_, purity ≥ 99.5%), ethyl acetate (C_4_H_8_O_2_, purity ≥ 99.5%), methanol (CH_4_O, purity ≥ 99.7%), ethanol (C_2_H_6_O, purity ≥ 99.7%), sodium hydroxide (NaOH, purity ≥ 99%), hydrochloric acid (HCl, 37%) and deionized water (H_2_O, 99.9%) were purchased from Sinopharm Chemical Reagent Co., Ltd. (Shanghai, China). Dialysis bag (molecular weight cutoff: 3000 Da) was purchased from Nanjing Dulai Biotechnology Co., Ltd. (Nanjing, China).

### 2.2. Preparation of GQDs

In this study, GQDs were prepared by the direct pyrolysis method, referring to a previous procedure [32] with a small amount of modification. Citric acid was used as the precursor, and the carbonization degree of citric acid was adjusted by controlling the reaction time and temperature to synthesize GQDs. The typical preparation process was as follows: 10 g citric acid was added to a flask, which was placed in an oil bath and heated to 200 °C for 30 min. When all the color of the solution had turned to pale yellow, the flask was taken out and cooled to room temperature. After that, 100 mL of NaOH solution with a concentration of 0.5 mol/L was dropped into the flask and stirred until the solution was evenly dispersed. Then, the pH value of the solution was adjusted to 7 using hydrochloric acid with a concentration of 2 mol/L. The obtained solution was further dialyzed for 48 h using a dialysis bag, for which the molecular weight cutoff was 3000 Da. Finally, the synthesized GQD solution was stored at 4 °C for further use.

### 2.3. Preparation of ZIF-8

ZIF-8 was prepared by a liquid-phase synthesis method [33] from the coordination of zinc ions with 2-methylimidazole. First, 1 mmol of zinc nitrate hexahydrate and 4 mmol of 2-methylimidazole were dissolved in 25 mL of methanol to prepare two types of solutions. The obtained solutions were stirred until the color turned transparent. Then, the methanol solution of 2-methylimidazole was poured into the methanol solution of zinc nitrate hexahydrate. The mixture was evenly dispersed, was left to stand for 24 h and was further washed via centrifugation at 10,000 rpm for 5 min. The centrifugal solvent had a 1:1 ratio of water and methanol. After three rounds of centrifugation, the residue powder was dried at 60 °C to obtain ZIF-8.

### 2.4. Preparation of GQDs@ZIF-8 Composites

GQDs@ZIF-8 composites were prepared from the combination of GQDs and ZIF-8 by a solution co-deposition method. First, 10 wt% of ZIF-8 powder was dissolved in methanol solution and dispersed with an ultrasonic cell pulverizer for 10 min. Then, 10 mg/mL of GQD solution was dropped into the prepared ZIF-8 solution, with a GQD content of 5%. After magnetic stirring for 30 min, the mixture was washed via centrifugation for 3 rounds, with a centrifugation time of 20 min and a rotating speed of 10,000 rpm. Then, the obtained samples were dried at 60 °C to obtain GQDs@ZIF-8 composites with a water content of zero.

### 2.5. Characterization of GQDs@ZIF-8 Composites

The crystal structure of the GQDs@ZIF-8 composites was measured by XRD (Shimadzu Limited, XRD-7000) with a scanning rate of 4°/min. The chemical structure was investigated by FT-IR (Shimadzu, IRSpirt) in the range of 400–4000 cm^−1^. The microstructure and morphologies were observed by TEM (JEOL, JEM 2100F). The components of the composite were determined by XPS (Thermo Scientific, K-Alpha). The thermal stability was investigated by TGA (Netzsch, TG 209F3) in the range of 25–800 °C at a heating rate of 5 °C/min. The Brunauer-Emmett-Teller (BET) N_2_ adsorption-desorption isotherms were measured by a surface area and porosimetry system (Quantachrome, NOVA) at 77 K to determine the specific surface area and pore structure of the composites.

### 2.6. Adsorption Test of GQDs@ZIF-8 Composites for Toluene and Ethyl Acetate

**Figure 1:**
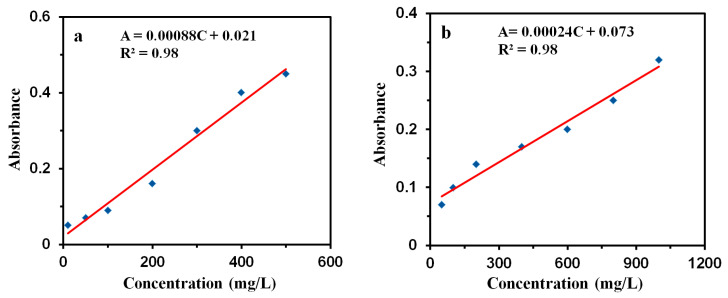
Standard working curves of toluene (**a**) and ethyl acetate solution (**b**). (The blue squares represent testing results, and the red lines represent fitting results).

Toluene and ethyl acetate were taken as the target VOC pollutants to determine the adsorption performance of GQDs@ZIF-8 composites. Different concentrations of toluene solution and ethyl acetate solution were prepared. UV-1800 ultraviolet spectrophotometer (Persee, TU-1810) was used to measure the absorbance of toluene at 261 nm and ethyl acetate at 256 nm. The absorbance-concentration curves of toluene and ethyl acetate were drawn to obtain the standard working curve, as shown in Figure 1. Then, the concentrations of the solution after each adsorption experiment were calculated based on the measured absorbance and the standard working curve.

Toluene solution with a concentration of 400 mg/L and ethyl acetate solution with a concentration of 800 mg/L were prepared. Different quantities of GQDs@ZIF-8 composites were put into the above solutions for the adsorption test. Moreover, the pH value of the solution was adjusted to 3, 5, 7, 9 and 11, respectively. The adsorption performance of the composites under different pH values was measured using an ultraviolet spectrophotometer and then the concentrations of toluene and ethyl acetate solution at different times were calculated according to the standard curve of the reference solution. The removal percentage R (%) and adsorption capacity Q_t_ (mg/g) of GQDs@ZIF-8 composites for toluene and ethyl acetate can be calculated using the following formulas:

(1) $$R=(C_{0}-C_{t})/C_{0}\cdot 100\%$$

(2) $$Q_{t}=(C_{0}-C_{t})\cdot V/m$$

where C_0_ is the initial concentration of the adsorbate solution (mg/L), C_t_ is the concentration of the adsorbate solution at time t (mg/L), V is the volume of adsorbate solution (L) and m is the dosing mass of the adsorbent material (g).

## 3. Results and Discussion

### 3.1. Morphology Analysis of GQDs@ZIF-8 Composites

**Figure 2:**
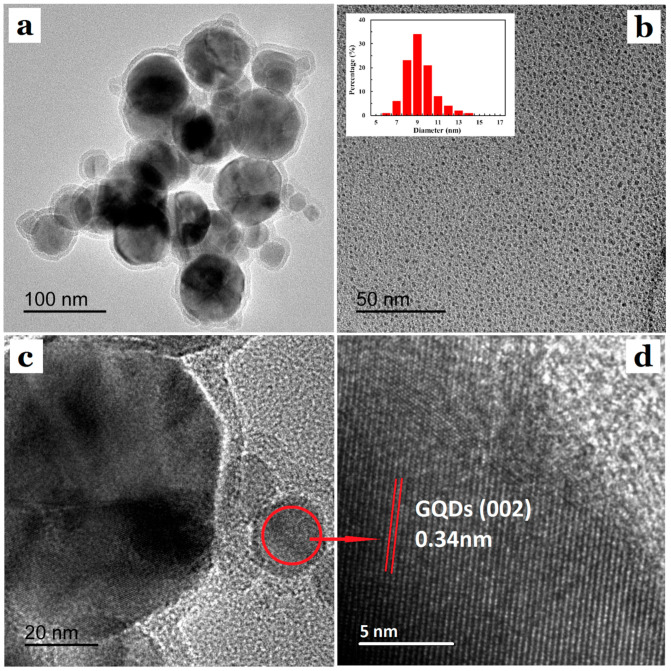
Morphology and microstructure images of GQDs@ZIF-8 composites. (**a**) TEM image of GQDs@ZIF-8, (**b**) TEM image of GQDs with diameter distribution graph, (**c**) HRTEM image of GQDs@ZIF-8, (**d**) HRTEM image of a signal GQD.

The morphology and microstructure images of GQDs@ZIF-8 composites are shown in Figure 2. From the TEM images of GQDs@ZIF-8 shown in Figure 2a, it can be seen that the composite presents a regular structure with a uniform distribution and tight connection. Figure 2b shows the TEM image of the GQDs, in which the insert picture is the particle size distribution diagram of the GQDs, revealing that the GQD particles are evenly distributed without obvious agglomeration. From the insert picture, it can be seen that the particle size distribution range of the GQDs is 5–15 nm. Figure 2c shows the HRTEM image of GQDs@ZIF-8, which reveals that GQDs@ZIF-8 exhibits an obvious core-shell structure, indicating that GQDs are successfully assembled on the surface of ZIF-8. The average particle size of the composite is approximately 90 nm. Figure 2d presents the HRTEM image of a signal GQD. It can be observed that the lattice stripe spacing of a single GQD is 0.34 nm, which corresponds to the (002) crystal plane of graphite. The morphology and microstructure analysis results prove the successful synthesis of GQDs@ZIF-8 composites. This core-shell structure of composites can provide enormous specific surface area and remarkably increase the active adsorption sites and, hence, improve the VOC adsorption capacity of the GQDs@ZIF-8 composite significantly.

### 3.2. Crystalline Structure and Chemical Bond Analysis of GQDs@ZIF-8 Composites

**Figure 3:**
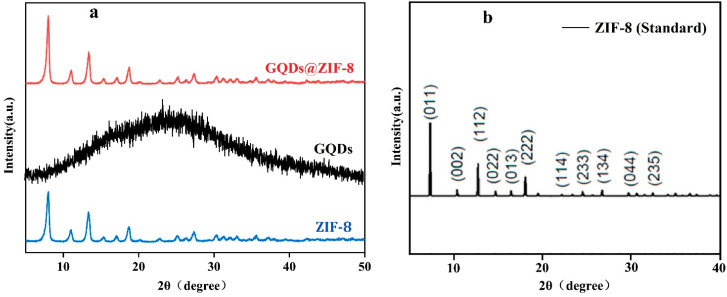
XRD patterns of GQDs, ZIF-8 and the GQDs@ZIF-8 composites. (**a**) Measurement results, (**b**) standard XRD pattern of ZIF-8. Adapted with permission from reference [34]. Copyright 2018, American Chemical Society.

Figure 3 exhibits the measured XRD pattern of the composites and the standard XRD pattern of ZIF-8. The XRD pattern of GQDs@ZIF-8 is similar to that of pure ZIF-8, and the diffraction peaks of ZIF-8 and GQDs@ZIF-8 are consistent with the standard peaks of ZIF-8 (Figure 3b) [34]. The 2θ values of the strong diffraction peaks are 7.3°, 10.3°, 12.7°, 14.7°, 16.4°, 18°, 24.6° and 26.7°, which correspond to the (011), (002), (112), (022), (013), (222), (233) and (134) planes of ZIF-8, respectively. In addition, the diffraction peak shape of GQDs@ZIF-8 changes due to the introduction of GQDs, and the pattern of the composite does not show the characteristic peak (25°) of the GQDs, which is due to the high dispersion and low content of GQDs in the composites. A similar phenomenon was also reported by another study on graphene oxide/ZIF-8 composites [35].

**Figure 4:**
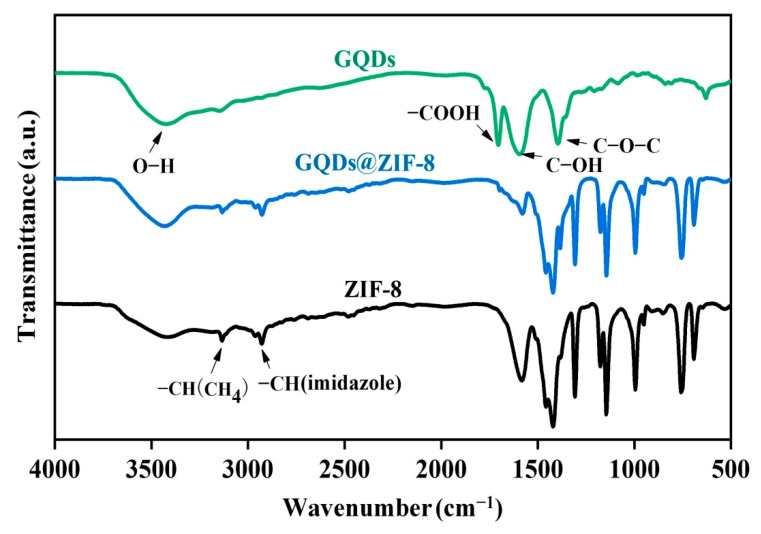
FT-IR spectra of GQDs, ZIF-8 and the GQDs@ZIF-8 composites.

The results of FT-IR spectroscopy in Figure 4 also confirm the interaction between ZIF-8 and GQDs in the GQDs@ZIF-8 composites. It is obvious that the infrared spectrum of GQDs@ZIF-8 is similar to that of ZIF-8. Most absorption bands of ZIF-8 and GQDs@ZIF-8 are related to the vibration of the imidazole unit. For example, the absorption peak at 1575 cm^−1^ is attributed to the stretching vibration of the C=N bond, the absorption peak in the range of 1350−1500 cm^−1^ is related to the stretching vibration of the imidazole ring and the strong absorption peaks at 1145 cm^−1^ and 994 cm^−1^ are attributed to the stretching vibration of the C−N bond in the imidazolyl group [36]. The infrared spectra of ZIF-8@GQD also reveal that it contains not only the absorption peaks of ZIF-8, but also the characteristic peaks from the GQDs, such as the stretching vibration of the C=O bond at 1728 cm^−1^ and the stretching deformation vibration of the C–OH bond at 1620 cm^−1^ [37]. These results confirm that GQDs are decorated on the structure of ZIF-8.

### 3.3. Chemical Components Analysis of GQDs@ZIF-8 Composites

**Figure 5:**
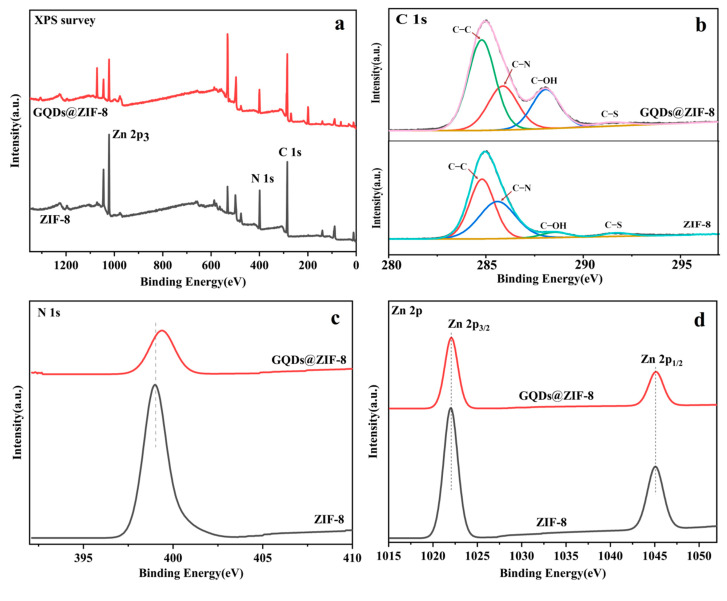
XPS survey spectra of ZIF-8 and GQDs@ZIF-8 composites (**a**), C 1s spectra with the colorful curves representing the characteristic peaks of C–C, C–N, C–O and C–S, respectively (**b**), N 1s spectra (**c**), Zn 2p spectra (**d**).

XPS analysis was performed to determine the elemental composition of the GQDs@ZIF-8 composites and the chemical states of specific elements, as shown in Figure 5. The full spectrum scans of ZIF-8 and GQDs@ZIF-8 are shown in Figure 5a, revealing that the characteristic absorption peaks of Zn 2p_3_, N 1s and C 1s appear at 1022.08 eV, 399.08 eV and 285.08 eV, respectively. The characteristic peaks demonstrate the presence of the elements necessary for the formation of GQDs@ZIF-8 [38]. The high-resolution C 1s spectrum in Figure 5b shows four different types of characteristic peaks of C–C, C–N, C–O and C–S, which correspond to 284.8 eV, 285.59 eV, 288.53 eV and 291.72 eV, respectively. In particular, due to the addition of GQDs, the C–S characteristic peaks representing GQDs appear in the spectrum of the GQDs@ZIF-8 composites. Figure 5c shows the symmetrical peak of N 1s at 399.8 eV corresponding to 2-methylimidazole [39,40]. Figure 5d reveals that the Zn 2p spectrum has two strong peaks at 1022 eV and 1045.07 eV, corresponding to 2p_3/2_ and 2p_1/2_, respectively [41]. When GQDs are loaded on the surface of ZIF-8, the position of the peak corresponding to Zn 2p shifts to the low binding energy region by 0.25 eV. This phenomenon indicates that, due to the modification of GQDs, the Zn oxidation degree is reduced, resulting in an increase in the electron density on Zn, which reveals that Zn is involved in the interaction of the two components [42]. XPS analysis confirmed the connection of the GQDs to ZIF-8.

### 3.4. Specific Surface Area and Pore Structure Analysis of GQDs@ZIF-8 Composites

**Figure 6:**
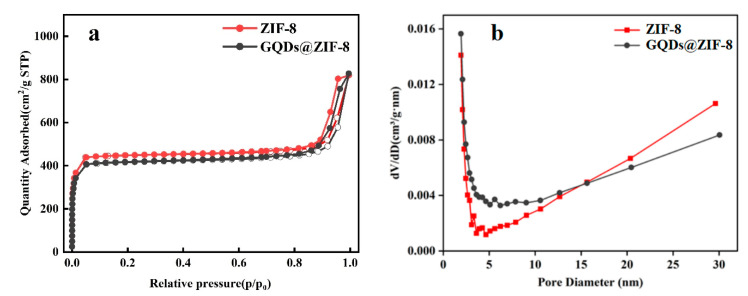
N_2_ adsorption–desorption isotherms with the hollow dots corresponding to adsorption process and the solid dots corresponding to desorption process (**a**), and pore size distribution (**b**) of ZIF-8 and GQDs@ZIF-8 composites.

The N_2_ adsorption-desorption isotherms and pore size distribution of the composites are shown in Figure 6. The ZIF-8 and GQDs@ZIF-8 samples showed isotherm curves of type I, with a significant upward process at relatively low pressures, indicating that micropores are the dominant structure in both ZIF-8 and GQDs@ZIF-8 composites. Meanwhile, the isotherm curve shows a slight secondary rise at higher pressure (when P/P_0_ = 0.9), indicating the existence of mesopores in the structure of the samples. The calculated specific surface area and pore structure parameters of ZIF-8 and GQDs@ZIF-8 are shown in Table 1. The specific surface area and average pore diameter of ZIF-8 are 1368.74 m^2^/g and 3.7 nm, respectively. After the addition of GQDs, the specific surface area of the GQDs@ZIF-8 composites decreases slightly to 1312.05 m^2^/g, with an average pore diameter of 3.9 nm, which reveals that the addition of GQDs can regulate the structure of GQDs@ZIF-8 composites. On the one hand, the increase in GQDs relatively reduces the number of pores per unit mass of the composite; on the other hand, GQDs attached to the surface of ZIF-8 can block the micropores of the composite. However, the pore size of the composites is close to that of ZIF-8, and it can be concluded that GQDs have been successfully introduced into the ZIF-8 structure, which is mutually confirmed by the above characterizations.

### 3.5. Thermal Stability Analysis of GQDs@ZIF-8 Composites

**Figure 7:**
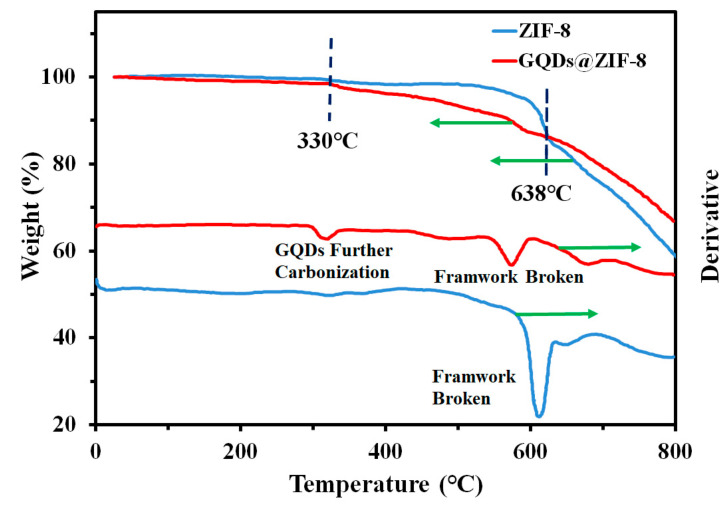
TG and DTG curves of ZIF-8 and GQDs@ZIF-8 composites, with the green arrows indicating the vertical coordinates corresponding to different curves.

The thermal stability of ZIF-8 and GQDs@ZIF-8 composites was tested by a TG analyzer to investigate the addition effect of GQDs. As shown in Figure 7, both samples showed high thermal stability. Before 330 °C, the samples only had slight weight loss, which is due to the evaporation of water molecules or the removal of other residual guest molecules. In sharp contrast, the weight loss rates of the two samples increased rapidly when the temperature reached approximately 600 °C, indicating that the skeleton structure of the samples was damaged and began to decompose. It is worth noting that the weight loss of GQDs@ZIF-8 is larger than that of ZIF-8 before 638 °C, whereas it is smaller than that of pure ZIF-8 after 638 °C. This is due to the further carbonization of ZIF-8 surface-loaded GQDs before 638 °C, resulting in a larger weight loss of the composite as a whole compared to pure ZIF-8, which is also reflected in Figure 7. After 638 °C, larger graphene sheets are generated from further carbonization of GQDs, covering the surface of ZIF-8 and protecting ZIF-8 from thermal decomposition. Thus, the residual weight of the composites is greater than that of pure ZIF-8. The above results reveal that GQDs improve the thermal stability of ZIF-8, which can expand the adsorption application of GQDs@ZIF-8 composites at a higher temperature.

### 3.6. Adsorption Performance of GQDs@ZIF-8 Composites for Toluene and Ethyl Acetate

**Figure 8:**
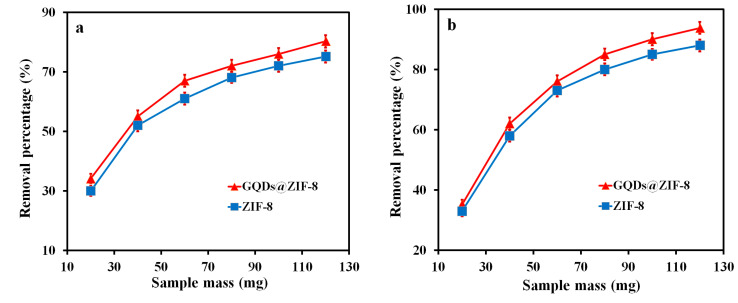
Effect of adsorbent mass on the removal percentage of toluene (**a**) and ethyl acetate (**b**).

Toluene and ethyl acetate solutions were prepared with concentrations of 400 mg/L and 800 mg/L, respectively, to test the adsorption performance of ZIF-8 and GQDs@ZIF-8 composites. A pH value of 7, adsorption time of 180 min and sample mass of 20, 40, 60, 80, 100 and 120 mg, respectively, were applied. It is worth noting that the GQD content of 5% was adopted to prepare GQDs@ZIF-8 composites, with the optimal adsorption effect. (Figure S1 in Supplementary Materials). Then, the effects of the adsorbent mass on the removal percentage of ZIF-8 and GQDs@ZIF-8 were investigated, as shown in Figure 8. With the continuous increase in the adsorbent mass, the removal percentages of toluene and ethyl acetate are significantly improved and finally tend to be stable and reach equilibrium. ZIF-8 and GQDs@ZIF-8 show strong adsorption ability for toluene and ethyl acetate in the early stage of adsorption. When the mass of GQDs@ZIF-8 composites increases from 20 mg to 120 mg, the removal percentage for toluene increases from 34.13% to 80.25%, while the removal percentage for ethyl acetate increases from 35.21% to 93.78%. Figure 8 clearly shows that the addition of GQDs obviously improves the removal percentage of ZIF-8, which increases from 75.13% to 80.25% when adsorbing toluene and from 87.94% to 93.78% when adsorbing ethyl acetate. In general, the removal percentage of porous materials for VOCs mainly depends on the specific surface area of the material. Although the specific surface area test results in Figure 6 show that the addition of GQDs slightly reduces the specific surface area of ZIF-8, GQDs@ZIF-8 exhibits a higher removal percentage for both adsorbates. This special adsorption phenomenon reveals the synergistic effect between GQDs and ZIF-8. The hydroxyl groups on the surface of GQDs and the imidazole groups in ZIF-8 can combine to form hydrogen bonds, resulting in the formation of new pore channels which increase the adsorption capacity of the composites for the adsorbate. In addition, GQDs with a large number of functional groups on the surface also provide active sites for toluene and ethyl acetate molecules, which are favorable for binding with adsorbate molecules. At the same time, since both GQDs and toluene molecules have benzene ring structures, the π–π bond interaction between them can also promote the adsorption of toluene [43].

**Figure 9:**
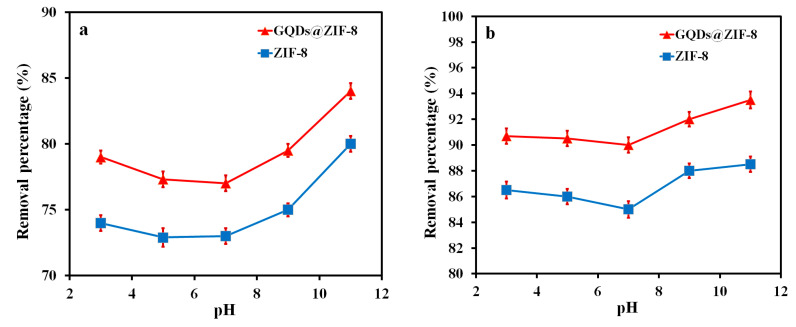
Effect of pH value on removal percentage of toluene (**a**) and ethyl acetate (**b**).

The influence of the pH value on the removal percentage of ZIF-8 and GQDs@ZIF-8 is shown in Figure 9. In this measurement, the sample mass of 100 mg, toluene concentration of 400 mg/L, ethyl acetate concentration of 800 mg/L, adsorption time of 180 min and pH value of 3, 5, 7, 9 and 11, respectively, were applied. It is obvious that both samples have a special response to the pH value of the toluene and ethyl acetate solution. Both acidic and basic conditions enhance the removal percentages of toluene and ethyl acetate, which is mainly due to the protonation and deprotonation of active sites at the edge of GQDs. As a kind of nanomaterial with a conjugated structure, GQDs contain abundant dissociable groups such as amino groups, carboxyl groups and hydroxyl groups on the surface, and the degree of dissociation directly depends on the pH value of the solution. The hydroxyl groups and imidazole groups in ZIF-8 can combine to form hydrogen bonds, which lead to the formation of new pore channels, thereby increasing the adsorption ability of the composites. In addition, the abundant functional groups on the surface also provide numerous active sites, which are favorable for binding with adsorbate molecules.

**Figure 10:**
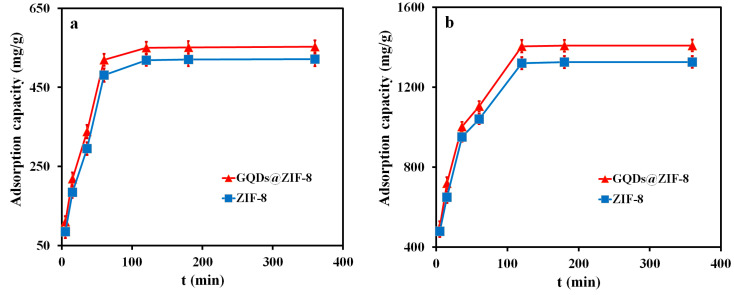
Effect of contact time on the adsorption properties of toluene (**a**) and ethyl acetate (**b**).

To investigate the influence of the contact time on the adsorption property of ZIF-8 and GQDs@ZIF-8 composites, the adsorption capacities of the adsorbent samples at different adsorption times (5, 15, 30, 60, 120, 180 and 360 min, respectively) were recorded and are shown in Figure 10. In this measurement, the sample mass of 100 mg, toluene concentration of 400 mg/L, ethyl acetate concentration of 800 mg/L and pH value of 7 were applied. It can be seen that, within 60 min of contact time, both the adsorption speed and adsorption capacity of the samples to toluene and ethyl acetate increases rapidly. When the contact time is 60 min, the adsorption capacities of ZIF-8 and GQDs@ZIF-8 for toluene are 480.73 mg/g and 519.56 mg/g, respectively. Meanwhile, the adsorption capacities of ZIF-8 and GQDs@ZIF-8 for ethyl acetate are 1040.56 mg/g and 1104.63 mg/g, respectively. When the contact time is longer than 120 min, the adsorption capacity of ZIF-8 and GQDs@ZIF-8 reaches saturation and remains unchanged. The maximum adsorption capacities of GQDs@ZIF-8 composites for toluene and ethyl acetate are 552.31 mg/g and 1408.59 mg/g, respectively. Compared to ZIF-8, the maximum adsorption capacities for toluene and ethyl acetate are increased by 53.82 mg/g and 104.56 mg/g, respectively. In addition, the curve also reveals that, in the absence of other variables, the optimal adsorption times of ZIF-8 and GQDs@ZIF-8 for toluene and ethyl acetate are both 120 min.

**Figure 11:**
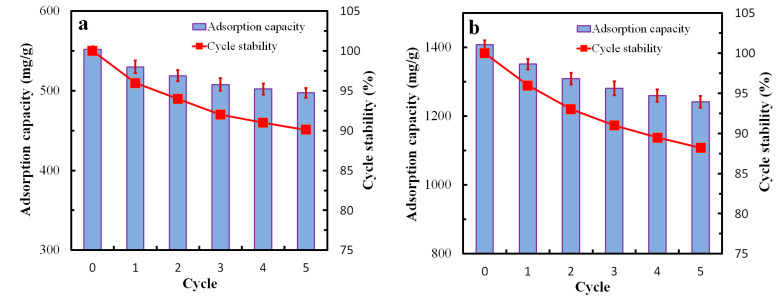
The adsorption–desorption cycle stability of the GQDs@ZIF-8 composite for toluene (**a**), and ethyl acetate (**b**).

To investigate the cyclic adsorption properties of the GQDs@ZIF-8 composites, five adsorption-desorption cycles were conducted, and the adsorption capacities after each cycle were measured, as shown in Figure 11. To carry out the desorption process, the samples that experienced adsorption were heated in a tube furnace to 150 °C for 1 h, with a N_2_ flow of 0.2 L/min as the protection gas, then the samples were taken out to obtain desorbed composites. The adsorption capacity of the GQDs@ZIF-8 composites for toluene in the initial cycle is 552.31 mg/g. After five cycles, the adsorption capacity is 497.35 mg/g, with a cycle stability (C_s_ = Q_t_/Q_1_) of 90.1%, while the adsorption capacity for ethyl acetate in the initial cycle is 1408.59 mg/g. After five cycles, the adsorption capacity is 1241.86 mg/g, with a cycle stability of 88.3%. The high cycle stability of the composites reveals the stable combination of GQDs and ZIF-8. The zeta potential analysis result also shows that GQDs and ZIF-8 have established a stable connection through strong electrostatic attraction effect (Table S1 in Supplementary Materials), which is consistent with this result. During the adsorption-desorption cycle, the nano-micro hierarchical porous structure of the composites makes it easier to adsorb and desorb dye molecules. In the first two cycles, the loss rate of the cycle stability is slightly higher, and then tends to be stable. This is because the desorption process was a heat treatment, which weakens the link between GQDs and ZIF-8 and reduces the cycle stability of the composites. However, even after five rounds of adsorption-desorption cycles, the adsorption capacity of the composites is around 90% of the initial level. Thus, the prepared GQDs@ZIF-8 composites have both excellent adsorption capacity and high cycle stability.

**Figure 12:**
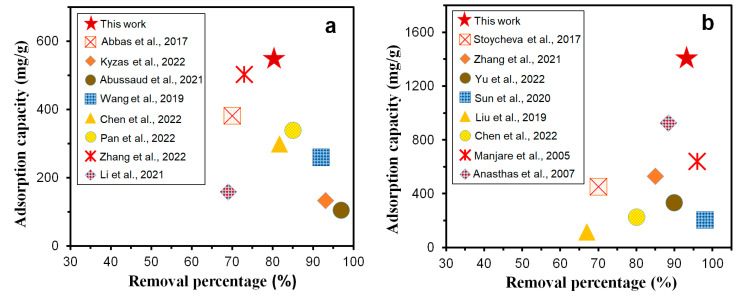
Comparison between previous works and this work on the adsorption properties of GQDs@ZIF-8 composites for toluene (**a**) [44,45,46,47,48,49,50,51] and ethyl acetate (**b**) [52,53,54,55,56,57,58,59].

The adsorption capacity and adsorption rate of previous adsorbents for toluene and ethyl acetate are shown in Figure 12 [44,45,46,47,48,49,50,51,52,53,54,55,56,57,58,59]. Compared with other adsorbents, the GQDs@ZIF-8 composites exhibit excellent performance both in adsorption capacity and in removal percentage due to their unique nano-micro hierarchical pore structures generated from the optimized matching of GQDs and ZIF-8. These structures not only possess a high specific surface area, but also maintain high cycle stability, as shown in Figure 11, which make the GQDs@ZIF-8 composite an ideal candidate for the highly efficient removal of VOCs.

### 3.7. Adsorption Kinetics Study of GQDs@ZIF-8 Composites

In order to further study the adsorption process of GQDs@ZIF-8 composites on VOC simulants (toluene solution and ethyl acetate solution), pseudo-first-order (PFO) kinetic models and pseudo-second-order (PSO) kinetic models were adopted to fit the experimental data according to the adsorption capacity of ZIF-8 and GQDs@ZIF-8 at different adsorption times. The PFO kinetic model and PSO kinetic model can be described by the following formulas [60,61,62]:

(3) $$\ln\left(Q_{e}-Q_{t}\right)=\ln Q_{e}-k_{1}t$$

(4) $$\frac{t}{Q_{t}}=\frac{1}{k_{2}Q_{e}{}^{2}}+\frac{t}{Q_{e}}$$

where Q_e_ is the equilibrium adsorption capacity (mg/g), Q_t_ is the adsorption capacity at time t (mg/g), k_1_ is the PFO kinetic constant (min^−1^) and k_2_ is the PSO kinetic constant (g/(mg/min)).

In addition, chi-square error (χ^2^) analysis [62] was adopted to evaluate the fitting quality by investigating the calculated results and experimental results, which can be described by the following equation:

(5) $$\chi^{2}=\sum_{i=1}^{n}{\left[\frac{{\left(Q_{e,\exp}-Q_{e,cal}\right)}^{2}}{Q_{e,cal}}\right]}_{i}^{}$$

where Q_e,exp_ and Q_e,cal_ represent the experimental and calculated values of the equilibrium adsorption capacity, respectively.

**Figure 13:**
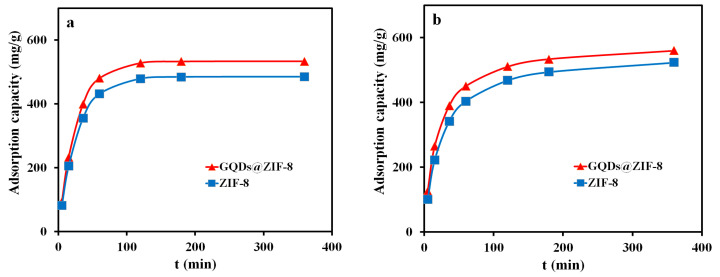
PFO (**a**), PSO (**b**) fitting curves of ZIF-8 and GQDs@ZIF-8 for toluene adsorption.

**Figure 14:**
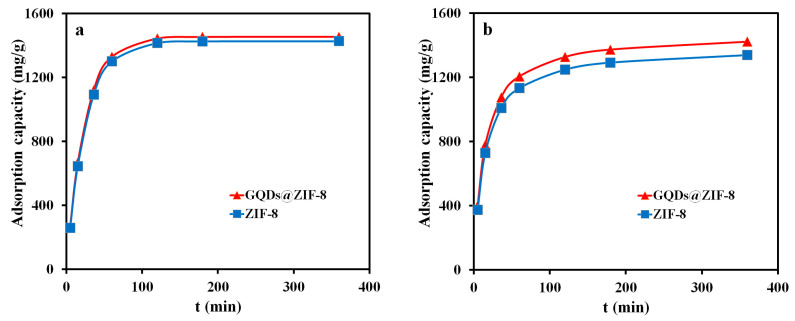
PFO (**a**), PSO (**b**) fitting curves of ZIF-8 and GQDs@ZIF-8 for ethyl acetate adsorption.

Based on the experimental results in Figure 10, the PFO model and PSO model of ZIF-8 and GQDs@ZIF-8 adsorption to toluene and ethyl acetate were fitted and are shown in Figure 13 and Figure 14. The corresponding kinetic parameters were calculated according to the fitting results, as shown in Table 2 and Table 3. In addition, the corresponding experimental conditions were as follows: the sample mass was 100 mg, the toluene concentration was 400 mg/L, the ethyl acetate concentration was 800 mg/L, the pH value was 7 and adsorption times were 5, 15, 30, 60, 120, 180 and 360 min, respectively.

Compared with the experimental results, the PFO and PSO model fitting results displayed good consistency with the experimental data, and the kinetic simulation also provided the theoretical adsorption capacity (Q_e,c_). After comparison, it was found that the experimental adsorption capacity of the GQDs@ZIF-8 composites is very close to the calculated adsorption capacity. The experimental adsorption capacity for toluene is 552.31 mg/g, while the PFO and PSO model calculation results are 533.57 mg/g and 588.23 mg/g, and the χ^2^ values are 0.66 and 2.19, respectively. In addition, when used for the adsorption of ethyl acetate, the experimental adsorption capacity of GQDs@ZIF-8 composites is 1408.59 mg/g, with PFO and PSO model calculation results of 1453.93 mg/g and 1474.31 mg/g and χ^2^ values of 1.41 and 2.93, respectively. Thus, based on the values of χ^2^ and R^2^ listed in Table 2 and Table 3, the adsorption processes of the GQDs@ZIF-8 composites are more consistent with the PFO model. The fitting results show that the adsorption process of the composites to the adsorbate molecules is mainly a physical adsorption process, including electrostatic adsorption generated by the surface functional groups and channel-filling adsorption generated by the nano-micro hierarchical porous structures in the GQDs@ZIF-8 composites.

### 3.8. Adsorption Isotherm Study of GQDs@ZIF-8 Composites

The Langmuir isotherm model and Freundlich isotherm model, which can be described by Equations (6) and (7), respectively [60,61,62], were used to investigate the adsorption isotherm processes.

(6) $$\frac{C_{e}}{Q_{e}}=\frac{1}{k_{L}Q_{m}}+\frac{C_{e}}{Q_{m}}$$

(7) $$\ln Q_{e}=\ln k_{F}+\frac{1}{n}\ln C_{e}$$

where C_e_ is the equilibrium concentration of the adsorbate solution (mg/L), Q_e_ is the equilibrium adsorption capacity (mg/g), Q_m_ is the maximum adsorption capacity (mg/g), k_L_ is the Langmuir constant (L/mg), k_F_ is the Freundlich constant and 1/n is the coefficient.

**Figure 15:**
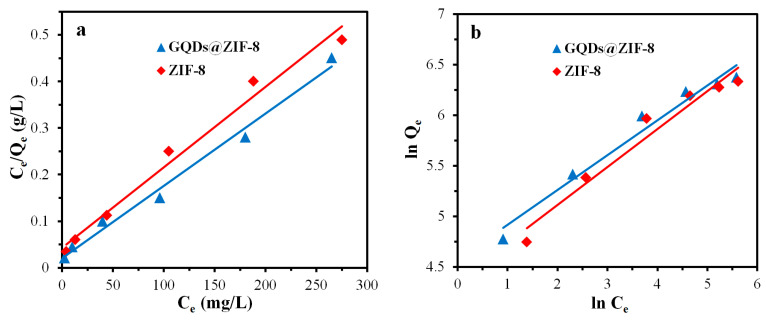
Langmuir model (**a**), Freundlich model (**b**) fitting curves of ZIF-8 and GQDs@ZIF-8 for toluene adsorption.

**Figure 16:**
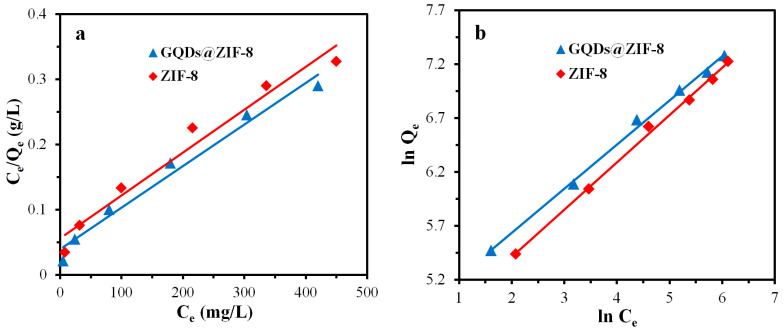
Langmuir model (**a**), Freundlich model (**b**) fitting curves of ZIF-8 and GQDs@ZIF-8 for ethyl acetate adsorption.

ZIF-8 and GQDs@ZIF-8 composites with a mass of 50 mg were immersed in toluene solutions with different concentrations of 50, 100, 200, 300, 400 and 500 mg/L to obtain the adsorption capacity of toluene. The composites with the same weight were immersed in ethyl acetate solutions with different concentrations of 100, 200, 400, 600, 800 and 1000 mg/L to obtain the adsorption capacity of ethyl acetate. After that, the fitting plots based on the Langmuir model and Freundlich model were drawn, as shown in Figure 15 and Figure 16, with the calculated equilibrium constants listed in Table 4 and Table 5, respectively. It can be seen from the fitting results that the theoretical maximum adsorption capacities of the composite for toluene and ethyl acetate are 628.93 mg/g and 1567.39 mg/g, respectively, while the experimental maximum adsorption capacities are 552.31 mg/g and 1408.59 mg/g, respectively, indicating that the experimental adsorption capacities of the composites are lower than the theoretical value. This comparison result reveals that the adsorption performance of the composite still has the potential to be further improved. Meanwhile, the fitting effect of the relevant linear coefficients of the Freundlich isotherm model is better, revealing that the adsorption of toluene and ethyl acetate molecules by the composite belongs to multi-molecular-layer adsorption, and the value of 1/n between 0.35 and 0.45 indicates that the adsorption process is relatively easy to realize.

### 3.9. Adsorption Thermodynamic Study of GQDs@ZIF-8 Composites

To carry out the adsorption thermodynamics study of the GQDs@ZIF-8 composites, the following equations [60,61] were adopted to investigate the thermodynamic parameters of the adsorption process:

(8) $$\Delta G=-RT\ln K_{d}$$

(9) $$\ln K_{d}=\frac{\Delta S}{R}-\frac{\Delta H}{RT}$$

(10) $$K_{d}=Q_{e}/C_{e}$$

where ΔG is the standard Gibbs free energy change (J/mol) in the adsorption process, ΔH is the standard enthalpy change (J/mol) in the adsorption process, ΔS is the standard entropy change (J/(mol·K)) in the adsorption process, R is the molar constant of gas, K_d_ is the adsorption equilibrium constant and T is the absolute temperature (K).

**Figure 17:**
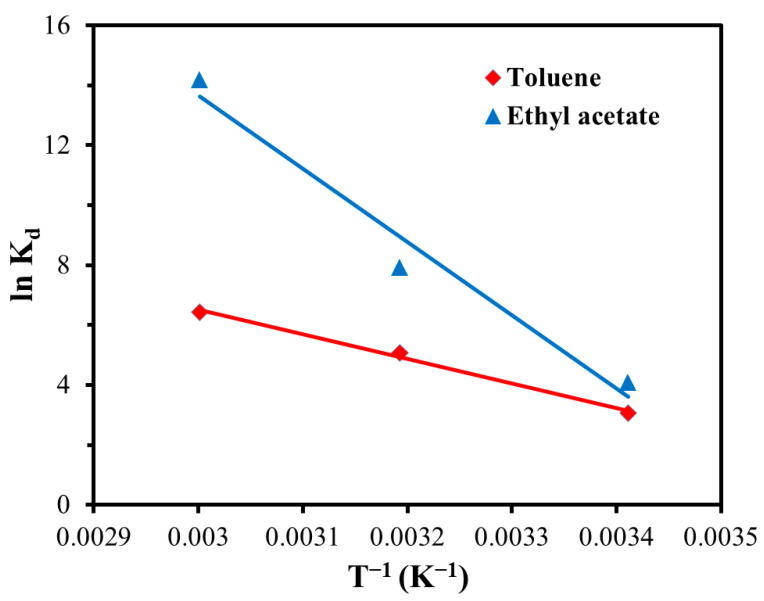
Thermodynamic fitting for the adsorption process of GQDs@ZIF-8 composites.

The adsorption tests were conducted at different temperatures of 20, 40 and 60 °C. GQDs@ZIF-8 composites with a mass of 50 mg were immersed in toluene solution with a concentration 400 mg/L and ethyl acetate solution with a concentration of 800 mg/L. After the measurement of adsorption properties at different temperatures, the thermodynamic fitting results for toluene and ethyl acetate adsorption processes were as shown in Figure 17, with the corresponding calculated thermodynamic parameters listed in Table 6 and Table 7. According to the results, all the values of ΔG < 0, which indicates that the adsorption process is spontaneous. The ΔH values of toluene and ethyl acetate adsorption processes are 32.65 KJ/mol and 58.45 KJ/mol, respectively, revealing that the adsorption processes are endothermic. In addition, the ΔS values of toluene and ethyl acetate adsorption processes are 132.23 J/(mol·K) and 223.98 J/(mol·K), respectively, revealing that the adsorption processes of the composites are at the entropy increase stage, accompanying the increase in the disorder degree both on the surface and inside the GQDs@ZIF-8 composites during the adsorption process.

**Figure 18:**
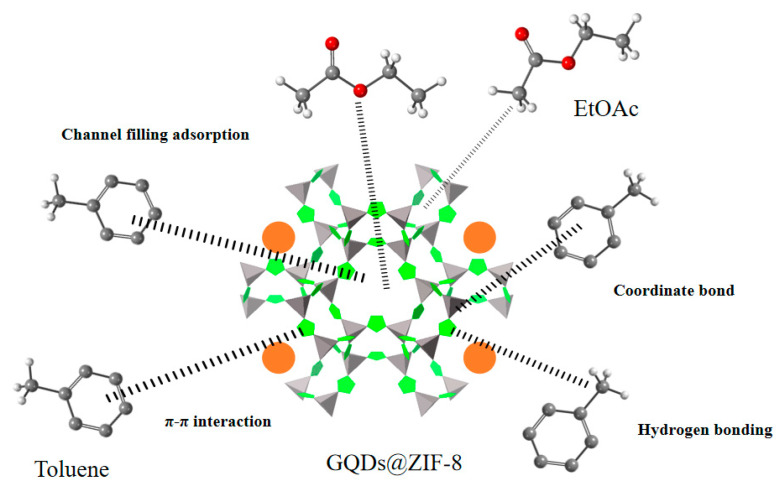
Adsorption mechanism illustration of the GQDs@ZIF-8 composites.

Based on the above investigation of GQDs@ZIF-8 composites, the possible adsorption mechanism of the composite is illustrated in Figure 18. The adsorption mechanism of the GQD/ZIF-8 composite for toluene and ethyl acetate mainly includes mesoporous channel filling, coordination bonding, hydrogen bonding and π–π interaction mechanisms [43,63]. The channel-filling mechanism fundamentally affects the adsorption performance of the composites. At the initial stage, the adsorbate molecules first diffuse rapidly on the outer surface of the GQDs@ZIF-8 composites, which is mainly achieved through physical adsorption, including macroporous adsorption and electrostatic adsorption on the surface of the composite. A large number of functional groups on the surfaces of GQDs and ZIF-8 can provide abundant active adsorption sites for toluene and ethyl acetate molecules. In the second stage, the adsorbate molecules gradually diffuse into the micropores of the composite and then are adsorbed and deposited in the inner wall of the micropores [64,65]. This stage is controlled by the diffusion rate, and the adsorption speed decreases gradually. In addition, the π–π bond interaction mechanism between the GQDs and toluene molecules can further enhance the adsorption effect of the GQDs@ZIF-8 composites.

## 4. Conclusions

In this work, GQDs and ZIF-8 were synthesized by the direct pyrolysis method and liquid-phase synthesis method, respectively. Then, a novel GQDs@ZIF-8 composite was obtained by introducing GQDs into the ZIF-8 structure using a solution co-deposition method. The prepared GQDs@ZIF-8 composites were further characterized by TEM, XRD, XPS, FT-IR, TG and N_2_ adsorption measurements to investigate the morphology, crystal structure, element composition and thermal stability of the composites. The results revealed that GQDs were uniformly decorated on the surface of ZIF-8 nanoparticles, effectively restraining agglomeration and improving the thermal stability of ZIF-8. The composites exhibit a large specific area of 1312.05 m^2^/g which provides abundant active sites by the synergistic effect between GQDs and ZIF-8. The influences of the adsorbent mass, contact time and pH value on the adsorption property of the composites were systematically investigated. When toluene and ethyl acetate were chosen as the target pollutants, the maximum adsorption capacity of GQDs@ZIF-8 composites reached 552.31 mg/g and 1408.59 mg/g, respectively, and the maximum removal percentages reached 80.25% and 93.78%, respectively. The kinetics and isotherm study revealed that the adsorption processes are in accordance with the pseudo-first-order kinetic model and the Freundlich isotherm model. The thermodynamic results indicated that the adsorption process of the GQDs@ZIF-8 composites is a spontaneous, endothermic and entropy increase process. To sum up, since the application of GQDs, ZIF-8 and their composites in the field of VOC adsorption has been explored, they have been widely used in the adsorption of various organic solvents, such as dichloromethane, acetone, toluene and ethyl acetate, etc. However, their adsorption properties need to be further improved to meet application requirements. This study further combined GQDs and ZIF-8, taking full advantage of the synergistic effect of microstructures, and obtained GQDs@ZIF-8 composites with excellent adsorption performance, which not only explored the application of GQDs@ZIF-8 composites in the adsorption field, but also provides a new method for the highly efficiency removal of VOCs.

## Figures and Tables

**Table 1:** Specific surface area and pore structure parameters of ZIF-8 and GQDs@ZIF-8.

Sample	S_BET_ (m^2^/g)	S_mic_ (m^2^/g)	V_tot_ (cm^3^/g)	V_mic_ (cm^3^/g)	D (nm)
ZIF-8	1368.74	1298.83	1.267	0.296	3.7
GQDs@ZIF-8	1312.05	1228.81	1.280	0.607	3.9

Note: S_BET_ is the specific surface area, S_mic_ is the micropore surface area, V_tot_ is the total pore volume, V_mic_ is the micropore volume, D is the average diameter of the pores.

**Table 2:** Adsorption kinetic parameters of ZIF-8 and GQDs@ZIF-8 for toluene adsorption.

Adsorbate	Experimental Adsorption Capacity Q_e,exp_ (mg/g)	Pseudo-First-Order Kinetic Equation				Pseudo-Second-Order Kinetic Equation			
		k_1_ (min^−1^)	Q_e,cal_ (mg/g)	R^2^	χ^2^	k_2_ (g/(mg·min))	Q_e,cal_ (mg/g)	R^2^	χ^2^
ZIF-8	521.02	0.036	485.46	0.99	2.60	0.000079	564.97	0.94	3.42
GQDs@ZIF-8	552.31	0.038	533.57	0.98	0.66	0.000093	588.23	0.95	2.19

**Table 3:** Adsorption kinetic parameters of ZIF-8 and GQDs@ZIF-8 for ethyl acetate adsorption.

Adsorbate	Experimental Adsorption Capacity Q_e,exp_ (mg/g)	Pseudo-First-Order Kinetic Equation				Pseudo-Second-Order Kinetic Equation			
		k_1_ (min^−1^)	Q_e,cal_ (mg/g)	R^2^	χ^2^	k_2_ (g/(mg·min))	Q_e,cal_ (mg/g)	R^2^	χ^2^
ZIF-8	1326.17	0.040	1388.88	0.99	2.83	0.000053	1425.53	0.94	6.93
GQDs@ZIF-8	1408.59	0.041	1453.93	0.99	1.41	0.000051	1474.31	0.97	2.93

**Table 4:** Calculated equilibrium constants of the isotherm model for toluene adsorption.

Adsorbent	Langmuir			Freundlich		
	Q_m_ (mg/g)	K_L_ (L/mg)	R^2^	K_F_ (L/mg)	1/n	R^2^
ZIF-8	588.23	0.047	0.95	78.34	0.38	0.98
GQDs@ZIF-8	628.93	0.057	0.94	96.57	0.35	0.98

**Table 5:** Calculated equilibrium constants of the isotherm model for ethyl acetate adsorption.

Adsorbent	Langmuir			Freundlich		
	Q_m_ (mg/g)	K_L_ (L/mg)	R^2^	K_F_ (L/mg)	1/n	R^2^
ZIF-8	1428.57	0.012	0.95	93.16	0.44	0.99
GQDs@ZIF-8	1567.39	0.016	0.96	123.66	0.41	0.99

**Table 6:** Calculated thermodynamic parameters for toluene adsorption.

T (K)	Thermodynamic Parameters		
	ΔG (KJ/mol)	ΔH (KJ/mol)	ΔS (J/(mol·K))
293.15	−6.09	32.65	132.23
313.15	−8.74		
333.15	−11.38		

**Table 7:** Calculated thermodynamic parameters for ethyl acetate adsorption.

T (K)	Thermodynamic Parameters		
	ΔG (KJ/mol)	ΔH (KJ/mol)	ΔS (J/(mol·K))
293.15	−7.18	58.45	223.98
313.15	−11.66		
333.15	−16.14		

## Data Availability

Not applicable.

## Supplementary Materials

The following supporting information can be downloaded at: https://www.mdpi.com/article/10.3390/nano12224008/s1, Figure S1: Effect of GQD content on the removal percentage of toluene and ethyl acetate; Table S1: ZETA potentials of GQDs, ZIF-8 and the GQDs@ZIF-8 composite.
