# Supplementary material for: Preparation and Adsorption Performance Study of Graphene Quantum Dots@ZIF-8 Composites for Highly Efficient Removal of Volatile Organic Compounds
Source: Nanomaterials (Basel). 2022 Nov 14;12(22):4008. doi: 10.3390/nano12224008 (PMC9695402; doi:10.3390/nano12224008)
Supplement: Supplementary file 1 [file nanomaterials-12-04008-s001.zip › nanomaterials-2010298-supplementary.pdf]

Preparation and Adsorption Performance Study of Graphene Quantum Dots@ZIF-8 Composites for Highly Efficient Removal of Volatile Organic Compounds

Hao Li [1,*], Youliang Cheng [2,*], Jiaxian Li [2], Tiehu Li [1], Jia Zhu [2], Weibin Deng [1], Jiajia Zhu [1] and Delong He [3]

1 School of Materials Science and Engineering, Northwestern Polytechnical University, Xi'an 710072, China
2 Faculty of Printing, Packaging Engineering and Digital Media Technology, Xi'an University of Technology, Xi'an 710048, China
3 Laboratoire de Mécanique Paris-Saclay, Université Paris-Saclay, CentraleSupélec, ENS Paris-Saclay, CNRS, 91190 Gif-sur-Yvette, France
* Correspondence: lihao@nwpu.edu.cn (H.L.); chengyouliang@xaut.edu.cn (Y.C.)

1. ZETA potential analysis

The ZETA potentials of the GQDs, ZIF-8 and the GQDs@ZIF-8 composites were measured by a zeta potential analyzer (Malvern, Zetasizer ZS90) in liquid phase without pH adjustment, and the results are shown in Table S1. The ZETA potentials of GQDs and ZIF-8 are -26.2 mV and 19.6 mV, respectively. After the compounding, the ZETA potential of the GQDs@ZIF-8 composite became 15.7 mV, revealing that GQDs was successfully grafted on the surface of ZIF-8, which was connected by strong electrostatic attraction with good stability.

Table S1. ZETA potentials of GQDs, ZIF-8 and the GQDs@ZIF-8 composite

	GQDs	ZIF-8	ZIF-8@GQDs
ZETA potential (mV)	−26.2	+19.6	+15.7

2. Effect of GQD content on the removal percentage of toluene and ethyl acetate

The influence of the GQD content (weight content of GQDs in GQDs@ZIF-8 composites) on the adsorption property of the GQDs@ZIF-8 composites was studied, with the GQD contents of 0, 3, 5 and 7%, respectively. The sample mass of 100 mg, toluene concentration of 400 mg/L, ethyl acetate concentration of 800 mg/L, adsorption time of 180 min, and pH value of 7 were applied. When the GQD content of 0% was applied, the raw ZIF-8 was obtained, as presented in Section 3.6 of the manuscript (Figures 8-10). When the GQD content was 5%, the adsorption property was the best. When the GQD content increased to 7%, the excessive GQDs trended to agglomerate on the surface of ZIF-8, causing the reduction of the adsorption property. Therefore, based on the experimental results, we chose a GQD content of 5% in this study.

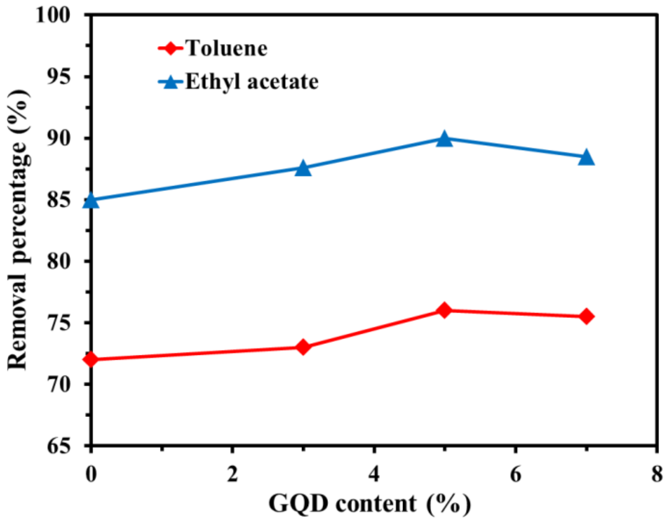

Figure S1. Effect of GQD content on the removal percentage of toluene and ethyl acetate
